# Identification of morphological and chemical markers of dry- and wet-season conditions in female *Anopheles gambiae* mosquitoes

**DOI:** 10.1186/1756-3305-7-294

**Published:** 2014-06-26

**Authors:** Kaira M Wagoner, Tovi Lehmann, Diana L Huestis, Brandie M Ehrmann, Nadja B Cech, Gideon Wasserberg

**Affiliations:** 1 Department of Biology, University of North Carolina Greensboro, 235 Eberhart Bldg, Greensboro, NC 27402, USA; 2 Laboratory of Malaria and Vector Research, National Institute of Allergy and Infectious Diseases, National Institutes of Health, 12735 Twinbrook Parkway, Rockville, MD 20852, USA; 3 Department of Chemistry and Biochemistry, University of North Carolina Greensboro, 435 Sullivan Bldg, Greensboro, NC 27402, USA

**Keywords:** Aestivation, Cuticular hydrocarbon, Photoperiod, Spiracle, Wing length, Malaria

## Abstract

**Background:**

Increased understanding of the dry-season survival mechanisms of *Anopheles gambiae* in semi-arid regions could benefit vector control efforts by identifying weak links in the transmission cycle of malaria. In this study, we examined the effect of photoperiod and relative humidity on morphologic and chemical traits known to control water loss in mosquitoes.

**Methods:**

*Anopheles gambiae* body size (indexed by wing length), mesothoracic spiracle size, and cuticular hydrocarbon composition (both standardized by body size) were examined in mosquitoes raised from eggs exposed to short photoperiod and low relative humidity, simulating the dry season, or long photoperiod and high relative humidity, simulating the wet-season.

**Results:**

Mosquitoes exposed to short photoperiod exhibited larger body size and larger mesothoracic spiracle length than mosquitoes exposed to long photoperiod. Mosquitoes exposed to short photoperiod and low relative humidity exhibited greater total cuticular hydrocarbon amount than mosquitoes exposed to long photoperiod and high relative humidity. In addition, total cuticular hydrocarbon amount increased with age and was higher in mated females. Mean n-alkane retention time (a measure of cuticular hydrocarbon chain length) was lower in mosquitoes exposed to short photoperiod and low relative humidity, and increased with age. Individual cuticular hydrocarbon peaks were examined, and several cuticular hydrocarbons were identified as potential biomarkers of dry- and wet-season conditions, age, and insemination status.

**Conclusions:**

Results from this study indicate that morphological and chemical changes underlie aestivation of *Anopheles gambiae* and may serve as biomarkers of aestivation.

## Background

Despite decades of intervention, malaria remains a primary global health concern, responsible for approximately 660,000 deaths per year [1]. Almost 90% of malaria deaths occur in Africa [2], where mosquitoes in the *Anopheles gambiae* Giles (*An. gambiae*) complex play a key role in the transmission of this disease [3]. In the Sahel region of Africa, malaria transmission is highly seasonal, reflecting availability of suitable larval sites for the vector [4]. Understanding the ecological processes that enable the persistence of *An. gambiae* in the Sahel could shed light on potential weak links in its life-cycle that could be used for mitigating malaria transmission in the region [5,6]. *An. gambiae* adults can be found only in small numbers during the Sahelian dry season, when surface water needed for larval development dries up for four to eight months [7]. However, population size appears to increase as much as ten-fold within seven days after the onset of rains [7]. Since *An. gambiae* eggs, larvae and pupae are non-resistant to seasonal desiccation [8-10] and since embryonic and larval development of *Anopheles* require at least nine days for adult emergence [11], this rapid population build-up cannot be explained by local population growth. Instead, these early wet-season adult mosquitoes are either migrating from surrounding areas with permanent water sources capable of harboring a continuously active population [5], or represent a local adult population that has undergone aestivation during the dry season and that emerge from their shelters following the first rains [6,7,12,13]. *Aestivation* is a dormant state associated with behavioral and physiological changes often leading to reproductive suppression and extended longevity of adult females in the dry-season [14,15]. Aestivation is considered an adaptation for survival during hot, dry periods, and has been observed in some insects, including the larva of the tiger moth *Cymbalophora pudica*[16], the pupa of Hessian fly *Mayetiola destructor*[17], and the adult alfalfa weevil *Hypera postica*[18]. While several studies have reported low-density presence of female Anopheline mosquitoes during the late dry-season [12,15,19,20], there is little hard evidence that rapid emergence of malaria vectors immediately after the onset of rains is a result of extended life by aestivation [7,20]. The recent recapture of one marked female *An. gambiae* at the end of the dry-season, 7 months after her release, confirmed that female aestivation does occur in Mali [7]. The extent to which adult aestivation contributes to *An. gambiae* population persistence in northern Mali has been investigated through comparison of wet-season population size of control sites (monitored only) and treatment sites receiving weekly dry-season pyrethrum sprays [6]. Wet-season *An. gambiae* (M-form) density was reported to be 30% lower in sites that received pyrethrum treatments [6], suggesting that adults were present during the dry-season, and thus that adult aestivation plays a significant role in *An. gambiae* dry-season survival and subsequent population buildup.

Suppression of water loss is a primary characteristic of species that face weather-induced desiccation and is expected to be important for the survival of *An. gambiae* during the dry-season in Mali. However, desiccation is also a constraint in winter diapausing mosquitoes, such as *Culiseta inornata*[21] and *Culex pipiens*[22]. In winter-diapausing *Culex pipiens*, three features were found to contribute to suppression of water loss: large body size, reduced metabolic rate, and increased quantity of cuticular hydrocarbons (CHCs) [22]. Desiccation has also been associated with increased length of saturated hydrocarbons (n-alkanes) in female *Drosophila melanogaster*[23], and with reduced spiracle size of *Anopheles stephensi*[24]. With respect to body size and CHC amount/n-alkane length, it is thought that reduction in water loss is the outcome of lower surface-area-to-volume ratio and reduced cuticle permeability, respectively [23,25,26]. Reduced metabolic rate and reduced activity, as well as related changes in size of spiracle openings, are thought to decrease insect respiratory water loss [27-31]. However, despite this and other evidence of physiochemical effects of desiccation in many mosquitoes and other insects [17,22-24,32,33], the morphological and chemical adaptations associated with aestivation have not yet been well evaluated in *An. gambiae*. In a recent study of *An. gambiae* in the Sahel, surprisingly, average wing length increased in the early dry season (December-February), decreased during the late dry season (April-May), and metabolic rate was not reduced during the dry-season as expected, but in fact peaked in the late dry-season [13].

The gaps in our understanding of the adaptations associated with Anopheline persistence throughout the Sahelian dry season, combined with unexpected results regarding wing-length and metabolism in supposedly aestivating populations, highlight the need for additional laboratory studies. Better understanding of the morphological and chemical characteristics that distinguish aestivating *An. gambiae* populations could contribute significantly to the development of novel strategies for vector control targeted at dry-season survivors [9]. Because it is unclear if the few *An. gambiae* found during the dry-season represent aestivators, morphological and chemical biomarkers such as CHC profiles may be useful for identification of aestivators. In this study, we tested the hypothesis that morphological and chemical changes compatible with aestivation are induced by short photoperiod and low relative humidity (SP-LRH), typical of the dry season in the Sahel. We predicted that larvae maintained under short photoperiods (SP) would develop into larger adults with decreased relative spiracle size when compared to larvae exposed to long photoperiods (LP). We also predicted that adults maintained under SP-LRH would exhibit increased total CHC amount and increased mean n-alkane length compared with those maintained under long photoperiod and high relative humidity (LP-HRH). We tested these predictions by comparing the morphologic traits, CHC composition and total CHC amount between *An. gambiae* reared in conditions that simulated the photoperiod and relative humidity (RH) of dry- or wet-season environments.

## Methods

### Experimental design

Mosquito rearing and experiments were conducted in insectaries at the Laboratory of Malaria and Vector Research, NIAID, NIH in the summer of 2010. The NIH-G3 colony, which originated in West Africa and was established in 1972 by the London School of Tropical Medicine, was used for all experiments. Dry- and wet-season conditions simulated in this experiment mimic dry- and wet-season conditions common to the Sahelian region of West Africa. Morphometric and gas chromatography–mass spectrometry (GC-MS) analyses of samples resulting from the experiment were conducted at the University of North Carolina at Greensboro.

#### Simulating dry- and wet-season conditions at the larval stage

At the larval stage, we simulated dry- and wet-season conditions by manipulating photoperiod, with shorter (11.5 hrs) daylength mimicking the dry season and longer (13.5 hrs) daylength mimicking the wet season. Daylength treatments were assigned randomly by tray and began with newly hatched larvae, which were reared in water-filled plastic pans and fed commercial fish food daily. Standard insectary conditions were maintained at 28°C, 75% relative humidity, and a 13.5-hour automatically timed photoperiod throughout the experiment. For wet-season conditions, the evening crepuscular period was mimicked by progressive darkening using an automatic timer (45 minutes in 15-minute intervals). For dry-season conditions, the evening crepuscular period needed to occur two hours earlier, and was therefore mimicked prior to the automatic dimming by progressive darkening of the containers using black cloth covers (two semi-transparent and the third opaque, added at 15 minute intervals). All treatments shared the same, automatically timed simulation of the morning crepuscular period. HOBO data loggers were used to monitor and record all temperature and relative humidity data. Morphological data (wing and spiracle length) was collected from 160 adult mosquitoes reared as larvae under dry- or wet-season photoperiods (n = 80 for each group). Adult mosquitoes were collected as they emerged over the first two days of emergence. Samples were placed into plastic screw-cap tubes containing 3-4 mL Drierite™ covered by cotton, then dried in an oven at approximately 55°C for 24 hours. Tubes were sealed with parafilm and frozen at -20°C until day of analysis.

#### Simulating dry- and wet-season conditions at the adult stage

**Figure 1:**
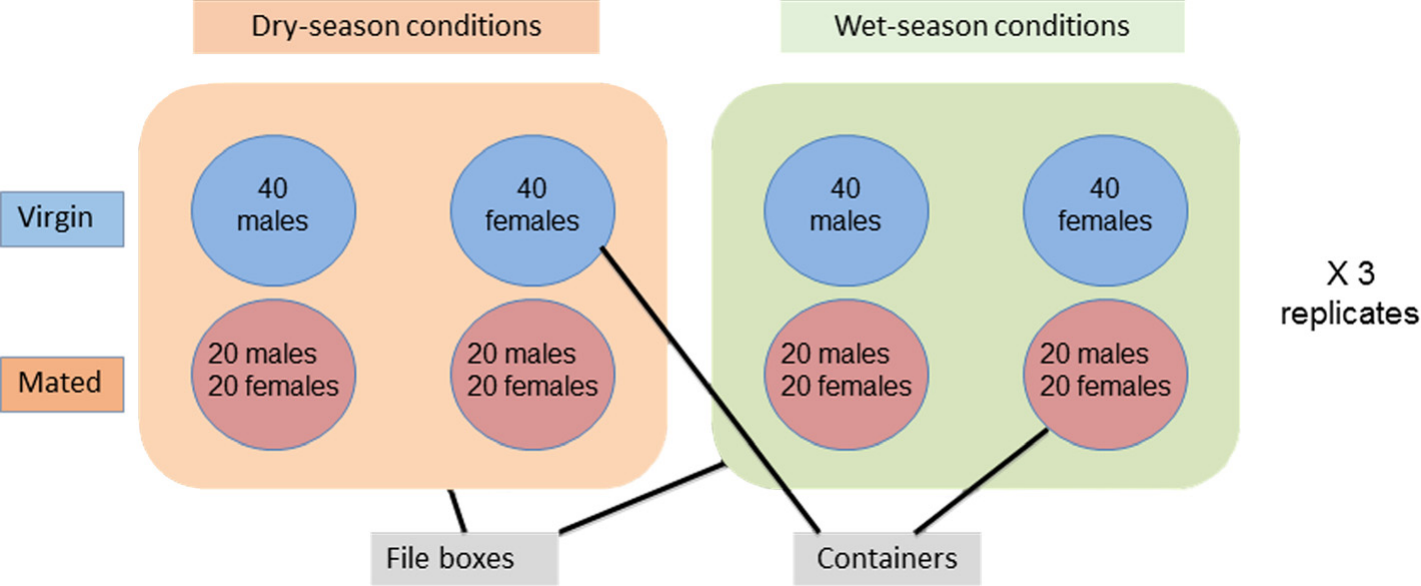
**Experimental setup for adult mosquitoes.** Large rectangles represent file boxes and circles represent 1-liter containers.

The daylight regimes outlined above were continued for adult mosquitoes used for cuticular hydrocarbon (CHC) analysis. In addition, adult relative humidity exposure was manipulated. Relative humidity (RH) treatment began on the first night after adult emergence. Adult mosquitoes were reared in cylindrical, 1 liter plastic containers covered with secured netting and placed inside transparent, air- and water-tight plastic file boxes (Ultimate File Box by Iris, IRIS-UCB-FB, 36.83 cm W × 45.47 cm L × 27.69 cm H) (hereafter, treatment boxes). For these treatment boxes, dry- and wet-season conditions were defined as 77% average RH with 11.5 hours of daylight, and 88% average RH with 13.5 hours of daylight, respectively. Dry-season humidity was induced using three 100 mm Petri dishes (per file box) containing 250 g each of Drierite™. Petri dishes were covered with non-airtight lids to slow moisture uptake by the Drierite™, which was replaced daily. Wet-season humidity was induced using 250 mL of a saturated NaCl solution placed inside each file box. Relative humidity in the containers was continuously monitored using HOBO data-loggers. To control for the effect of sex and mating status, adult mosquitoes were separated on the day of their emergence into female, male, or mixed-sex groups, using a mouth aspirator. It was assumed that female mosquitoes in the first group were non-mated and that mosquitoes in the third group would become inseminated. Four containers were placed into each file box: 1 male-only (n = 40), 1 female-only (n = 40), and 2 with both males and females (n = 20 of each sex) (Figure 1). Secured netting covered each container, such that mosquitoes separated into virgin and mated groups were exposed to identical simulated ambient conditions. Adult mosquitoes were given access to both water and a 10% sugar solution-soaked cotton ball that was changed daily. CHC data was collected from these mosquitoes. To account for the effect of adult age and mating status on CHC characteristics, we sampled mosquitoes from each container over time: Twelve females were randomly collected from each of the two treatment conditions (dry- and wet-season) on day 1, and four mosquitoes were randomly collected from each container on days 4, 9, 14, and 19 post-emergence. For mated mosquitoes, this sample of four was divided into two males and two females. In total, 108 mosquitoes were collected for CHC analysis, half reared under each treatment. Samples were preserved as described above.

### Sample analysis

#### Measurements of body size and spiracle length

**Figure 2:**
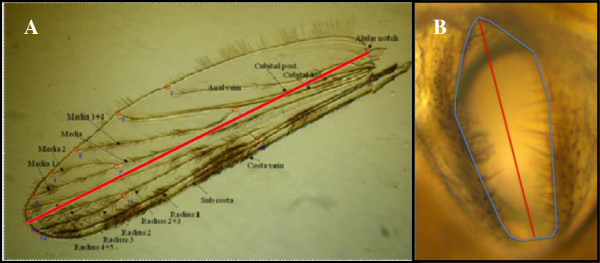
**Wing and spiracle landmarks. A)** Wing Landmarks (25x). Photo by Lehmann Lab, with permission, Laboratory of Malaria and Vector Research, NIAID, NIH. **B)***An. gambiae* spiracle (40x). Outer line indicates boundaries of spiracular opening, inner line indicates transverse diameter (spiracle length).

Response variables in morphological analysis were wing length and spiracular index (SI). Wing length was used as a measure of body size [34]. We mounted each wing on a microscope slide using Euparal Mounting Medium (BioQuip Products Inc.), photographed it using a microscope camera (Lumenara, model: Infinity 1) on an Olympus CX41 microscope (35× magnification), and measured its length using Infinity-Capture Software (Lumenara Inc.). Wing length was defined as the distance between the alular notch and the intersection of the radius 3 vein and the outer margin [35] (Figure 2A). The lengths of both wings were measured and the average length was recorded and used for analysis. To measure spiracle size, mosquitoes were pinned to an entomological needle that was stabilized on a threaded nut filled with modeling clay. Pictures of the mesothoracic spiracles of each mosquito were taken (40× magnification) and measured using Infinity-Capture Software. The opening of the spiracle is ellipsoid, and spiracle length was measured as the distance across the spiracle’s major axis (Figure 2B). The spiracle length value used for data analysis was the average (right and left) spiracle length per individual. Given the positive association between spiracle size and body size (r^2^ = 0.45, p < 0.001), spiracle size was standardized by dividing the spiracle length for each individual by that individual’s mean wing length, referred to hereafter as spiracular index or SI [24].

#### Cuticular hydrocarbon analysis

Response variables of the chemical analysis included standardized total cuticular hydrocarbon quantity (tCHC), mean n-alkane retention time (t_R_a), and standardized quantities of individual CHC peaks (iCHC). Following wing and leg removal, single specimens were submerged in 15 μl of heptane for 15 minutes at room temperature. Wing and leg removal facilitated CHC extraction by decreasing the volume of heptane needed to fully submerge each specimen. For each specimen, 2 μl of sample was injected into the GC-MS for analysis. Use of heptane rather than hexane prevented extract evaporation during operation of the GC-MS’s auto-sampler. Samples were analyzed on a Shimadzu GC-MS-QP2010S (operating at 0.97 kV and acquiring m/z values from 50 to 550). Source and interface temperatures were 200 and 330°C respectively. A 30 m RTX-5 column with 0.25 mm diameter, 0.5-μm stationary phase thickness was used with helium as the carrier gas (column head pressure 71.8 kPa, flow rate of 0.73 ml/m, linear velocity as the flow control mode). Injection temperature was 280°C and injection mode was ‘splitless’. After a 1 minute hold, the oven temperature rose from 75 to 160°C at 15°C/min, and then from 160 to 320°C at 5°C/min, with a final hold at 320°C for 20 min. Only the 15 largest (by % area), and reproducibly quantifiable peaks were examined for quantitative variability. Mass spectral libraries used for peak identification included NIST 2005 and WILEY 2007, including supplementary editions. GC-MS post-run analysis software calculated match percentage using an algorithm that compared spectra of the unknown compounds with statistically significant ions from known library spectra. CHC length determination was based on comparison with an external standard composed of Supelco n-hydrocarbon mix (even-numbered alkanes from C8 to C40, diluted 1000:1 with heptane) and spiked with pentadecane (C15). Total CHC quantity was calculated for each mosquito as the sum of the area under the 15 largest peaks. Given the positive association between body size and total CHC (r^2^ = 0.12, p = 0.032), total CHC was standardized by dividing the total CHC area for each individual by that individual’s mean wing length. The standardized total cuticular hydrocarbons are referred to hereafter as tCHC. Retention time reflects n-alkane chain length and, when combined with CHC quantity data, can be used as a measure of hydrocarbon relative abundance. Thus, mean n-alkane retention time (t_R_a) was calculated as the weighted mean of all n-alkane peaks (peaks 3, 5, 6, 9, 13, and 14) by multiplying n-alkane peak area by retention time, taking the sum, and then dividing by the total n-alkane area for that mosquito [23]. Standardized CHC quantities of individual peaks were calculated by dividing the area of peaks at specific retention times by mean wing length (as described above), and are referred to hereafter as iCHC.

#### Data analysis

To analyze the effect of the photoperiod treatment (simulated dry- and wet-season conditions) and development time (a covariate) on morphological characteristics (wing length and SI), a two-way Analysis of Variance (ANOVA) was used. For CHC analysis, peak area and retention times were averaged for sets of two mosquitoes from the same treatment box, container (insemination status), and age (collected on the same day). To analyze the effect of treatment, age, and insemination status on tCHC, t_R_a, and iCHC, we used split-split-plot ANOVA. Use of split-split plot analysis allowed dry- and wet-season treatments to be applied at the treatment box level, and insemination treatment to be applied at the container level [36]. In order to maintain consistency for comparisons between individual peaks, all factors, including non-significant ones, were kept in the models for analysis of iCHC. Additional 2-way ANOVAs were used to analyze the effect of climatic conditions and age on tCHC for virgin and mated individuals separately.

## Results

### Morphological markers

#### Wing length

**Figure 3:**
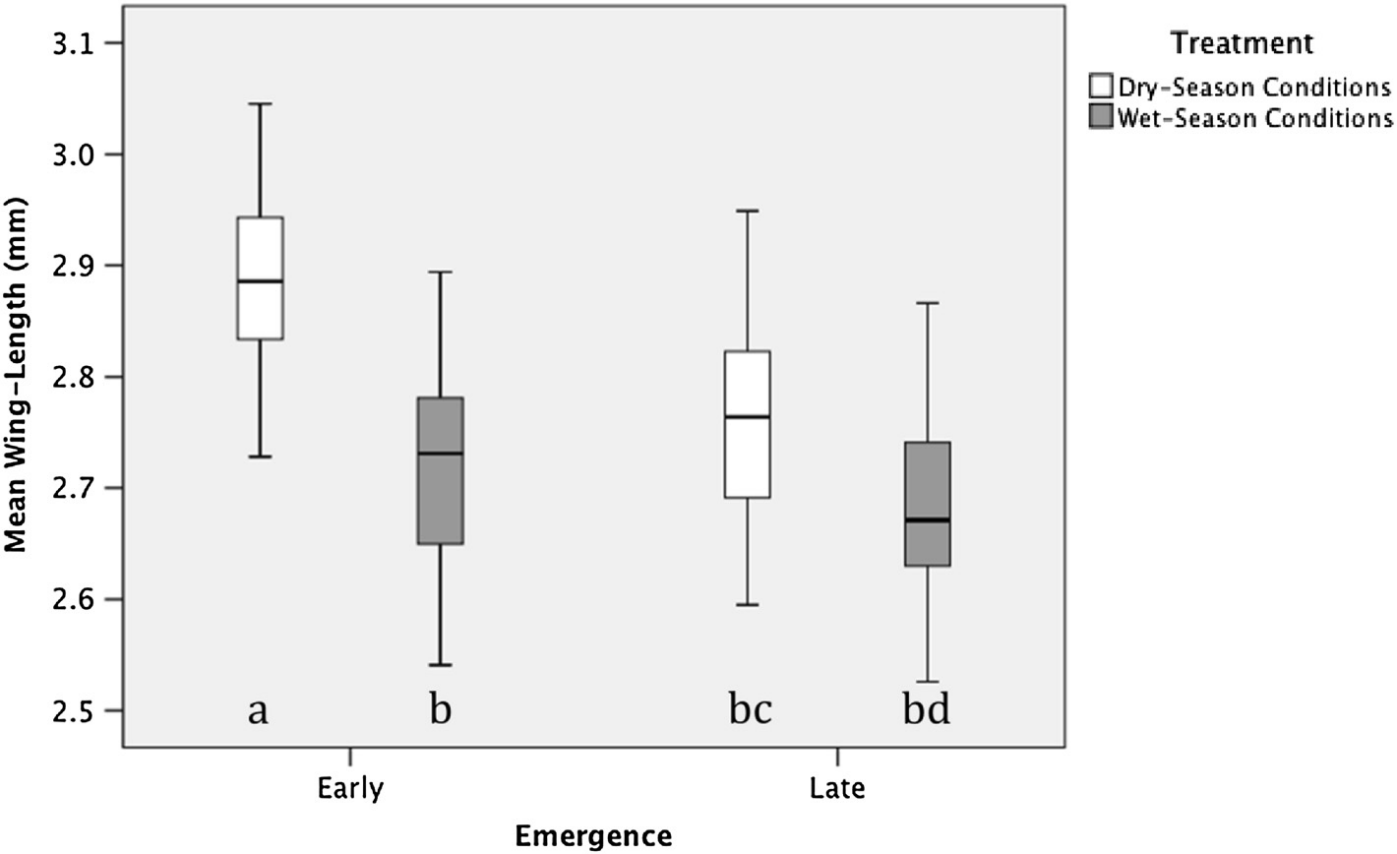
**Effect of photoperiod and development time on *An. gambiae *wing length, with 95% confidence intervals.** Different letters indicate significant (p < 0.05) pairwise differences between the four treatment by development time groups, as determined by post hoc Tukey HSD comparisons.

Consistent with our expectations, photoperiod treatment had a significant effect on wing length (Table 1), with mean wing length of SP mosquitoes (simulating the dry season) estimated to be 4.4% greater than that of LP mosquitoes (simulating the wet season) (Figure 3). Development time was also significantly associated with wing length (Table 1), with mean wing length of early emerging mosquitoes estimated to be 2.9% greater than that of mosquitoes emerging on day 2 (Figure 3). As indicated by the significant interaction between photoperiod and emergence day (Table 1), the effect of photoperiod treatment was greater for mosquitoes that emerged on day 1 (Figure 3).

**Table 1:** **Effect of photoperiod and emergence day on *An. gambiae *** **wing length**

**Source**	**Coefficient**	**SE**	**t**	**Sig.**
(Constant)	2.878	0.014	210.195	<0.001
Photoperiod	-0.156	0.020	-7.987	<0.001
Emergence day	-0.115	0.019	-5.922	<0.001
Photoperiod × emergence day	0.075	0.027	2.745	0.007

Multiple regression model, R^2^ = 0.407.

#### Spiracle length

**Figure 4:**
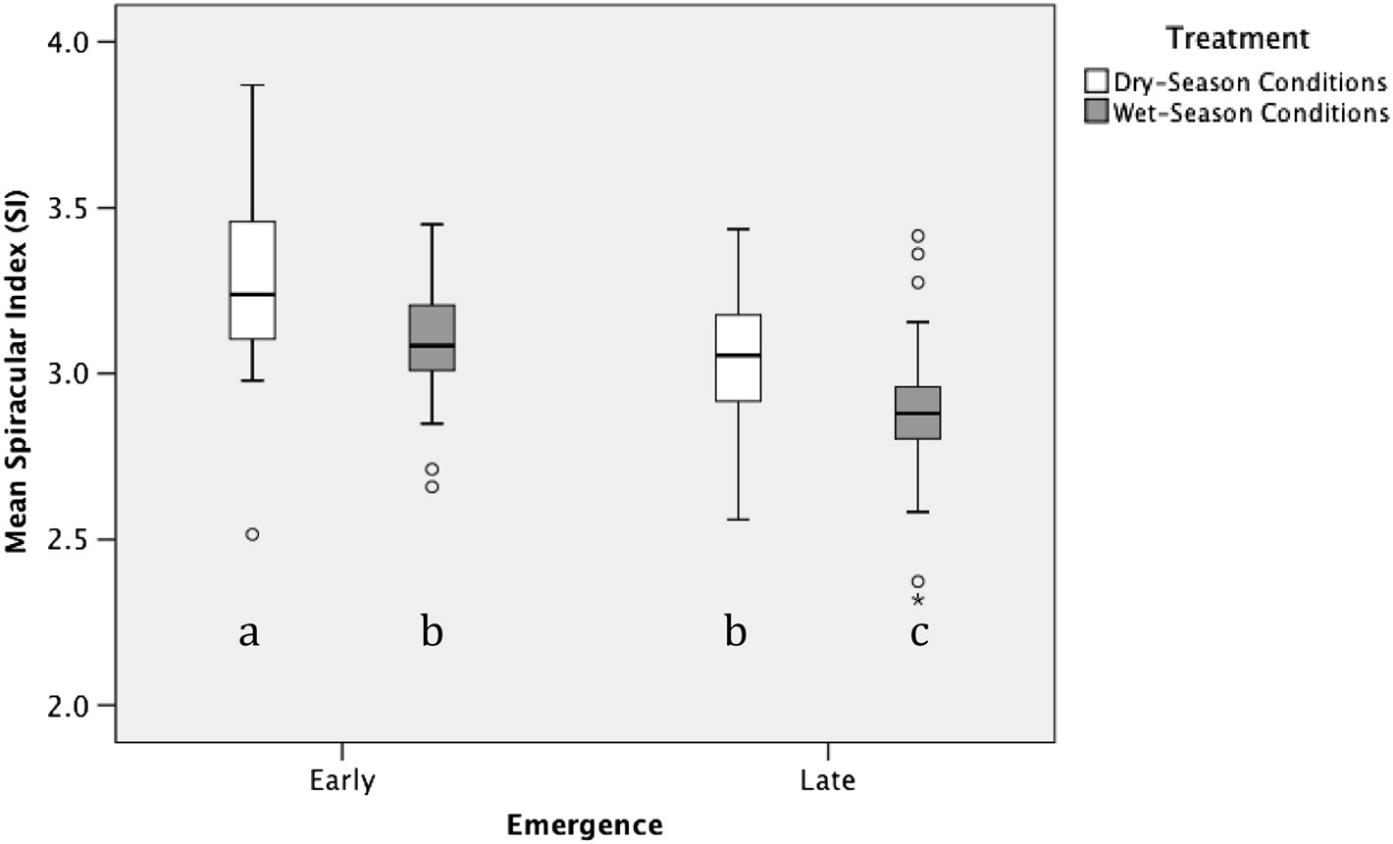
**Effect of photoperiod and emergence day on *An. gambiae *spiracular index, with 95% confidence intervals.** Different letters indicate significant (p < 0.05) pairwise differences between the four treatment by development time groups, as determined by post-hoc Tukey HSD comparisons.

Photoperiod treatment had a significant effect on SI, but in the opposite direction of our original expectation (Table 2). Mean SI of SP mosquitoes was 5.5% greater than that of LP mosquitoes (Figure 4)*.* Early emergence was also associated with a significant increase in SI (Table 2) with mean SI of the earliest-emerging mosquitoes estimated to be 7.3% greater than that of mosquitoes emerging on day 2 (Figure 4). No significant interaction between photoperiod and development time was detected (t = 0.101; df = 149; p = 0.92, data not shown).

**Table 2:** **Effect of photoperiod and emergence day on *An. gambiae *** **spiracular index**

**Source**	**Coefficient**	**SE**	**t**	**Sig.**
(Constant)	3.26	0.03	110.39	<0.001
Photoperiod	-0.16	0.03	-4.73	<0.001
Emergence day	-0.22	0.03	-6.31	<0.001

Multiple regression model, R^2^ = 0.297.

### Chemical markers

#### Standardized total CHC quantity

**Figure 5:**
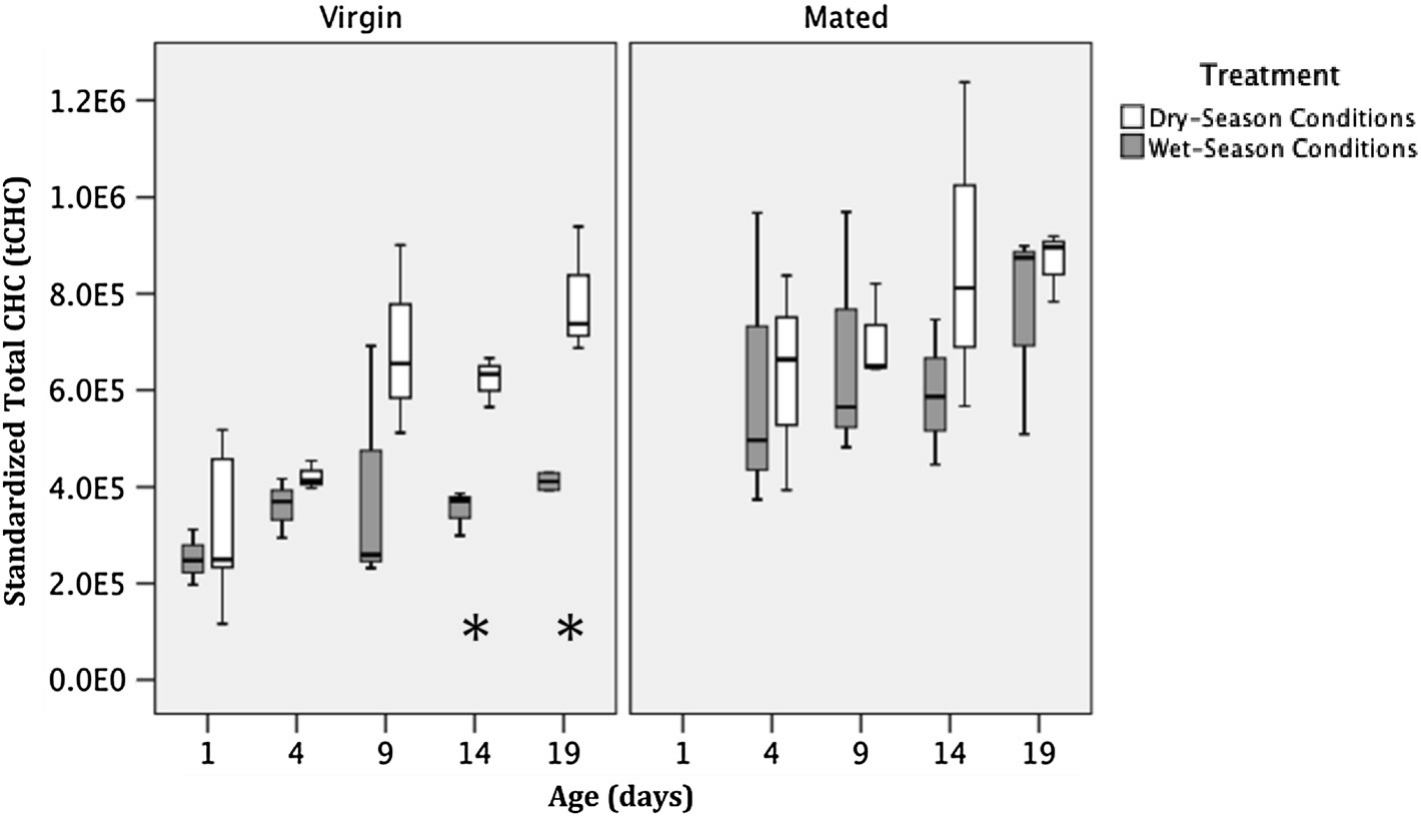
**Effect of treatment, age and insemination status on total (standardized) *An. gambiae *cuticular hydrocarbons.** Asterisks represent a significant difference (t-test, p ≤ 0.05) between treatments for each age group.

The effect of treatment (photoperiod and humidity combined) on tCHC was statistically significant (Table 3). On average, females reared under dry-season conditions (SP-LRH), had 28% greater tCHC than females reared under wet-season conditions (LP-HRH). The tCHC increased significantly with age (Table 3), with females aged 19 days having, on average, 152% more tCHC than newly emerged females (Figure 5). Insemination had a marginally significant effect (Table 3), with inseminated females having 58% greater tCHC than virgin females (Figure 5). No significant statistical interactions between the three factors were found. However, it appears that the effect of the treatment tends to be larger at older ages (Figure 5). Also, when analyzed separately, the effect of treatment was significant in virgin individuals (F = 18.4; df = 1; p < 0.001) but not significant in mated individuals (F = 1.4; df = 1; p = 0.26) (Figure 5).

**Table 3:** Effect of treatment conditions, insemination status, and age on total standardized CHC quantity

**Source**	**SS**	**df**	**MS**	**F**	**p**
Treatment	2.99E + 11	1	2.99E + 11	6.30	0.05
Insemination	4.78E + 11	1	4.78E + 11	5.52	0.07
Age	2.58E + 11	3	8.60E + 10	4.24	0.01

Split-split plot ANOVA.

#### N-alkane mean retention time

**Figure 6:**
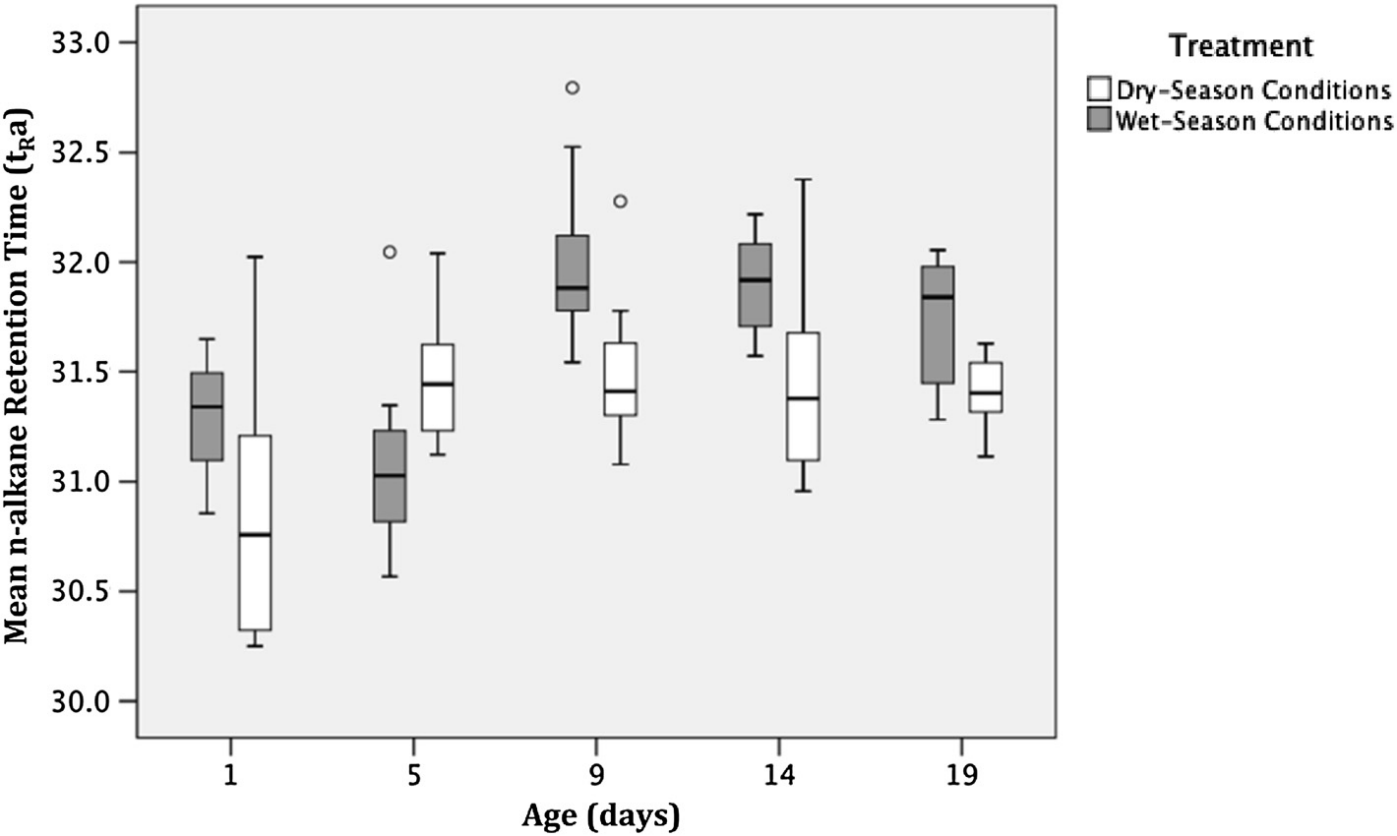
**Effect of treatment and age on *An. gambiae *** **mean n-alkane retention time.**

In contrast with our prediction, mosquitoes reared under LP-HRH had a significantly higher mean n-alkane retention time (t_R_a) than those reared under SP-LRH conditions (Table 4, Figure 6). Similar to the tCHC, t_R_a increased with age (Table 4, Figure 6). However, as indicated by the significant interaction between age and treatment, the effect of treatment seems to differ with age (Figure 6). The effect of insemination status on t_R_a was non-significant (F = 0.28; df = 1; p = 0.38, data not shown).

**Table 4:** **Effect of treatment conditions, age, and their interaction on *An. gambiae *** **mean n-alkane retention time**

**Source**	**SS**	**df**	**MS**	**F**	**p**
Treatment	0.91	1	0.91	9.01	0.04
Age	1.54	3	0.51	4.62	0.007
Age × treatment	1.63	3	0.54	4.91	0.005

Split-split plot ANOVA.

#### Peak identification

The hydrocarbons corresponding to peaks 3, 5, 6, 8, 9, 10, 12, 13, and 14 were tentatively identified based on fragmentation spectra matches in the NIST and WILEY libraries (Table 5). The most likely structural assignments from among the possible matches were chosen based on a literature review of mosquito cuticular hydrocarbons [22,37-41], and a comparison of retention times with an external hydrocarbon standard. Peak identifications include 6 n-alkanes, 2 monomethyl alkanes, and 1 dimethyl alkane (Table 5).

**Table 5:** Individual peak identification with approximate retention time (in minutes) and percent library match

**Peak #**	**Approximate RT**	**Identification**	**Formula**	**Library match**
**1**	23.9	-	-	-
**2**	24.0	-	-	-
**3**	25.3	Pentacosane	C_25_H_52_	94%
**4**	26.2	-	-	-
**5**	28.3	Heptacosane	C_27_H_56_	94%
**6**	29.7	Octacosane	C_28_H_58_	88%
**7**	30.1	-	-	-
**8**	30.7	1-Hexacosene	C_26_H_52_	84%
**9**	31.0	Nonacosane	C_29_H_60_	96%
**10**	31.4	2,3-dimethylnonadecane	C_21_H_44_	81%
**11**	31.9	-	-	-
**12**	33.4	9-Hexacosene	C_26_H_52_	94%
**13**	33.6	Hentriacontane	C_31_H_64_	79%
**14**	34.0	Dotriacontane	C_32_H_66_	92%
**15**	34.3	-	-	-

#### Individual CHC peak quantities

**Figure 7:**
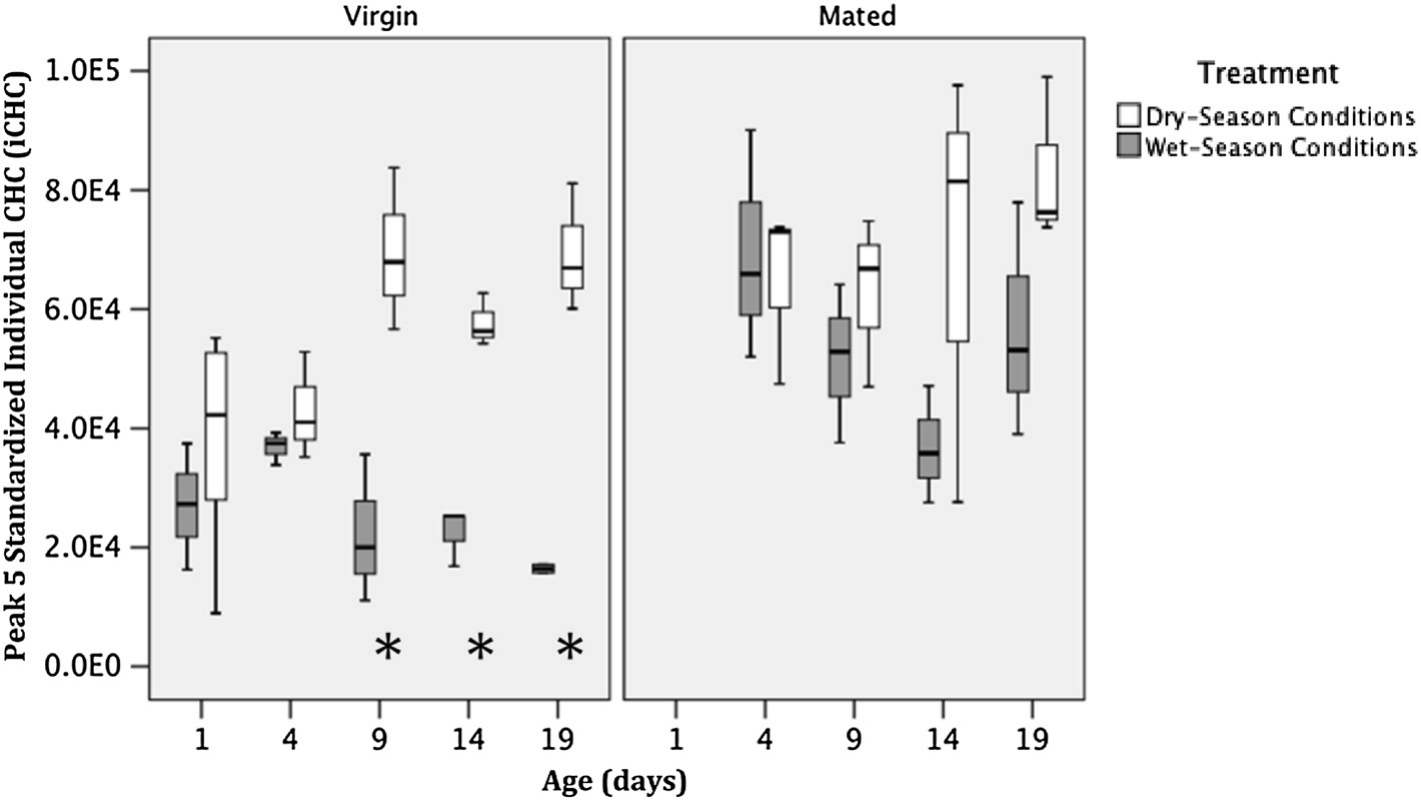
**Effect of treatment, age and insemination status on (standardized) *An. gambiae *peak 5 quantities.** Asterisks represent a significant difference (t-test, p ≤ 0.05) between treatments for each age group.

A significant treatment effect was found for three standardized individual cuticular hydrocarbon (iCHC) peak areas, with mosquitoes reared under SP-LRH conditions having significantly higher iCHC areas for peaks 3, 5 and 11 (Table 6). Also, a significant effect of mating was found for six iCHC peaks, with mated mosquitoes having significantly higher areas for peaks 2, 3, 5, 9, 10 and 14 (Tables 5 and 6). Peak 10 was the only structurally identified peak that increased significantly for mated individuals but was not affected by other variables (Table 6). A significant age effect was observed for peaks 6, 9, 13 and 15 (Table 7). In peaks 3 and 5, there were significant treatment-by-age interactions, where iCHC increased significantly with age only in mosquitoes raised under dry-season conditions (Table 7, Figure 7).

**Table 6:** Effect of treatment conditions and insemination on mean standardized individual peak CHC quantities

**Peak**	**Dry-season**	**Wet-season**	**Mated**	**Virgin**
1	24,160 ± 997	23,515 ± 1,466	27,395 ± 1,080	21,034 ± 1,027
2	9,777 ± 512	9,141 ± 474	**10,809 ± 401**	**8,421 ± 465**
3	**19,487 ± 1,896**	**11,328 ± 2,229**	**20,836 ± 2,433**	**11,608 ± 1,658**
4	9,562 ± 812	9,003 ± 878	11,509 ± 775	7,538 ± 726
5	**60,406 ± 3,842**	**39,311 ± 3,971**	**61,931 ± 4,248**	**41,607 ± 3,706**
6	4,171 ± 529	3,263 ± 583	5,383 ± 608	2,445 ± 376
7	***18,162 ± 1,088***	***16,290 ± 1,213***	20,177 ± 1,086	14,990 ± 1,007
8	7,817 ± 897	5,189 ± 1,046	9,247 ± 1,258	4,483 ± 511
9	***82,023 ± 9,865***	***61,093 ± 8,171***	**95,370 ± 9,024**	**53,904 ± 8,101**
10	9,636 ± 1,007	7,362 ± 1,009	**11,704 ± 1,186**	**6,086 ± 593**
11	**20,040 ± 1,377**	**16,720 1,501**	22,383 ± 1,194	15,238 ± 1,347
12	***13,164 ± 1,528***	***10,093 ± 1,835***	16,308 ± 2,047	7,915 ± 910
13	54,122 ± 4,838	46,192 ± 5,905	66,661 ± 4,983	37,482 ± 4,249
14	***79,468 ± 6,586***	***62,428 ± 6,372***	**95,254 ± 6,197**	**52,639 ± 4,557**
15	223,964 ± 15,945	171,392 ± 16,283	242,412 ± 17,235	165,396 ± 13,544

Significance of effect is based on split-split plot ANOVA, where bold pairs indicate significant (p < 0.05) effect of treatment or mating status and bold italic pairs indicate marginally significant (0.05 < p < 0.09) effect of treatment or mating status.

**Table 7:** Effect of age on mean standardized individual peak CHC quantities

**Peak**	**Treatment**	**1 Day**	**4 Days**	**9 Days**	**14 Days**	**19 Days**
1	All	21,451 ± 2,700	23,773 ± 1,822	29,083 ± 3,200	22,183 ± 1,557	24,439 ± 1,417
2	All	6,107 ± 986	9,767 ± 810	10,736 ± 837	10,124 ± 450	10,487 ± 711
3^†^	Aestivating	11,738 ± 5,528	17,746 ± 4,620	20, 328 ± 2,469	22,857 ± 6,093	23,229 ± 2,317
	Non-Aestivating	2,621 ± 923	24,790 ± 5,046	9,509 ± 3,719	4,596 ± 1,676	10,490 ± 2940
4	All	4,480 ± 1,162	9,663 ± 1,476	9,967 ± 1,316	9,719 ± 816	12,342 ± 1,121
5^†^	Aestivating	37,409 ± 8,579	53,778 ± 6,554	67,316 ± 4,461	63,360 ± 9,912	76,335 ± 5,509
	Non-Aestivating	27,013 ± 6,138	53,180 ± 8,766	36,354 ± 8070	29,652 ± 4,288	46,657 ± 12,875
6	All	**1,337 ± 199**	**2,728 ± 602**	**4,137 ± 822**	**3,802 ± 858**	**6,710 ± 846**
7	All	12,333 ± 2,820	17,272 ± 1,997	18,028 ± 1,594	18,166 ± 1,191	20,817 ± 1,447
8	All	4,191 ± 828	8,791 ± 2,228	7,242 ± 1,489	5,386 ± 1,382	7,524 ± 1,374
9	All	**12,768 ± 2,418**	**40,970 ± 4,451**	**72,619 ± 8,848**	**94,911 ± 11,071**	**136,113 ± 10,799**
10	All	4,755 ± 981	9,197 ± 1,869	9,383 ± 1,523	7,444 ± 1,379	12,362 ± 1,806
11	All	11,261 ± 2,900	18,455 ± 2,562	20,782 ± 1,999	19,280 ± 1,420	21,548 ± 2,018
12	All	6,532 ± 1,050	15,955 ± 3,521	13,581 ± 3,205	9,709 ± 2,193	12,481 ± 2,412
13	All	**16,130 ± 3,158**	**36,558 ± 5,643**	**56,933 ± 7,477**	**59,371 ± 5,982**	**80,757 ± 6,093**
14	All	25,415 ± 4,396	73,379 ± 9,264	88,905 ± 12,049	72,810 ± 9,136	93,543 ± 9,812
15	All	**124,982 ± 23,858**	**164,668 ± 17,612**	**244,254 ± 29,301**	**216,786 ± 28,658**	**245,004 ± 16,750**

Significance of effect is based on split-split plot ANOVA, where bold indicates significant (p < 0.05) effect of age and ^†^cross indicates a significant (p < 0.05) treatment-by-age interaction.

## Discussion

The primary goal of this study was to evaluate whether *An. gambiae* females respond differentially and adaptively to environmental cues characteristic of the dry- vs. wet-seasons. As expected for adaptation to dry ambient conditions [22-24], we predicted that emerging adults exposed as larvae to short, dry-season photoperiods would be characterized by larger body size and reduced spiracle size compared with larvae exposed to long, wet-season photoperiods. Similarly, we predicted that newly emerged adult females exposed to dry-season conditions (shorter day-length and lower relative humidity) would possess a higher total amount of CHCs and longer-chain CHC molecules compared with those exposed to wet-season conditions (longer day-length and higher relative humidity). As expected, and consistent with observations from the early dry season in Mali [13], larval exposure to dry-season photoperiods induced an increase in body size of newly emerged adults relative to those exposed to the wet-season photoperiods. However, in contrast to our expectation, larval exposure to dry-season photoperiods induced relatively larger (standardized) spiracles in these young adult females as compared with wet-season females. As expected, under dry season conditions, the total amount of CHCs increased; however, in contrast with the expectations, shorter CHC molecules were more abundant. Standardized individual CHC amounts varied with seasonal treatment, age and insemination status.

### Morphological markers

Insect cuticular water loss decreases inversely with surface area-to-volume ratio, such that larger insects are more resistant to desiccation [23,29,42]. Hence, adult mosquitoes reared as larvae under a short (dry-season) photoperiod were expected to be larger compared with mosquitoes reared under a long (wet-season) photoperiod. Indeed, under a shorter photoperiod, mosquitoes were 4.4% larger (measured as wing length), consistent with previous studies linking large body size to dry conditions [13,22,42,43]. For example, Lanciani [43] found wing length of *An. quadrimaculatus* to be 5.1 to 9.4% greater in females exposed to artificial short photoperiods (8:16 hours light:dark compared with 16:8 hours light:dark). Size of adults has also been associated with other environmental factors such as temperature, food availability, larval density, and presence of predators [44-50]. However, because these variables were controlled for in our experiment, the difference in body size can be attributed to the effect of the photoperiod to which they were exposed.

We also found that adult body size of the fastest-developing larvae was, on average, 2.9% larger than that of slower-developing larvae (Figure 3). Particularly interesting was the observation that the effect of photoperiod on adult body size was stronger for fast-developing as compared with slow-developing larvae (Figure 3). Fast-developing larvae may represent individuals that are able to exploit resources quicker and therefore emerge earlier with a larger adult body size [51-53]. The weaker effect of photoperiod on slow-developing larvae may reflect the fact that these mosquitoes were smaller to begin with.

Since water is lost through the spiracles during insect respiration [54], we expected that, as an adaptation against desiccation, spiracle length would be smaller in mosquitoes about to enter the dry-season [27-31,54]. In contrast to our prediction, standardized spiracle length was larger in mosquitoes reared under dry-season conditions (Figure 4). It is possible that, since energy is required to close the thoracic spiracles [31], longer but narrower spiracles may be selected for under dry-season conditions to facilitate efficient gas exchange with reduced energy expenditure. To evaluate a possible micro-scale morphological adaptation providing reduced spiracular water-loss, we are currently conducting a scanning electron-microscope analysis of spiracle size variation including traits such as spiracle width and depth, as well as the density and length of surrounding setae/scales.

### Cuticular hydrocarbons as seasonal markers

In this study, we demonstrated that, after standardizing for body size, the total amount of the 15 most-abundant CHCs was significantly greater for mosquitoes reared under dry-season conditions (Figure 5). This result is consistent with our hypothesis of the role of increased tCHC in reducing trans-cuticular water loss. Similar results have been reported by others [22,55]. For example, Benoit & Denlinger [22] observed a twofold increase in CHCs for winter diapausing *Cu. pipiens,* which was associated with a 30% reduction of water loss in these mosquitoes. However, since they did not standardize for body size, it is unclear whether this increase in CHC amount was independent of the increase in body size. Similarly, Urbanski *et al.*[55] reported that diapausing eggs from a temperate population of *Aedes albopictus* had one-third more surface CHCs and one half the water-loss rates as compared with non-diapausing eggs. In contrast, researchers did not find differences in CHC quantities when comparing Drosophila species from messic and xeric regions [29]. Our study did not evaluate the adaptive effect of CHC amount in *An. gambiae* in terms of reduced water loss; however, a follow-up study is planned.

In addition to the effect of simulated dry-season conditions on total CHC amount, we also observed an increase in total CHC amount with mosquito age (Figure 5). Similarly, Benoit & Denlinger [22] determined that CHC buildup at the adult stage is a flexible trait. They observed that the total amount of CHCs of *Cu. pipiens* increased with adult age during the first 10 days after emergence, and then leveled off. They also demonstrated faster CHC buildup in mosquitoes undergoing photoperiod-induced diapause [22]. Furthermore, they demonstrated a reduction in adult CHC quantity by changing from a diapausing to a non-diapausing photoperiod 40 days post-emergence [22]. In our study, total CHC increased continuously during the 19 days of the experiment for mosquitoes from both treatment groups, though the rate of increase was higher for mosquitoes reared under dry-season conditions. More importantly, the observation that 1-day-old virgin females reared under dry- or wet-season conditions do not differ in their total CHC amount suggests that differences in photoperiod during the larval stage do not induce differences in total CHC amount of eclosing young adults. Whether the cue for differential rates of CHC buildup is related to differences in relative humidity or photoperiod is impossible to disentangle, as the mosquitoes in this study were exposed to a combination of both. Further study of this issue is planned.

The effect of mating status on total CHCs was marginally significant, with CHC amount tending to be higher in mated females. A similar effect of mating status on the CHC profile has been demonstrated previously in Drosophila [56] and *An. gambiae*[40]. We believe that the marginally significant effect observed here is an underestimate, since insemination rate of *An. gambiae* in the laboratory has been found to be consistently low (72% maximum) [57], and since our experimental set-up did not guarantee that all females in the mixed-sex containers were mated.

Contrary to our prediction, mean CHC length was shorter in mosquitoes reared under simulated dry-season conditions compared with those reared under simulated wet-season conditions (Figure 6). It is possible that production of shorter CHCs allows for an increase in total CHC quantity, and that total CHC quantity is more important than hydrocarbon length for suppression of trans-cuticular water loss. It is also possible that smaller n-alkanes compact better, and are therefore more effective at sealing the cuticle. The relative abundance of longer-chain CHCs increased with age as predicted, which is consistent with findings from *An. gambiae, Aedes aegypti*[40], and *An. farauti*[39]. This pattern, however, differed among the treatment groups, with average CHC chain length for dry-season mosquitoes starting low, increasing sharply between 1 and 5 days old, and stabilizing thereafter, while for wet-season mosquitoes, average chain-length starts longer, increases until day 9, and stabilizes thereafter. Interestingly, the trend suggesting that 1-day-old mosquitoes reared as larvae under dry-season photoperiod eclose with shorter CHC molecules compared with those reared under wet-season photoperiod suggests the possibility that photoperiod is an important cue in determining the initial CHC composition of young adult female mosquitoes.

While mosquitoes reared under dry-season conditions appear to differ with respect to body size, spiracle length, total CHC amount, and n-alkane length, all of these traits co-vary with other environmental factors. Under natural conditions, uncontrolled environmental factors could mask these climatic effects. Therefore, identifying specific compounds that are indicative of dry- or wet-season conditions could be useful for identifying the time of the year when a particular mosquito has eclosed. For example, mean CHC amount of the unidentified peak 11 differed between dry- and wet-season conditions and was not affected by age or insemination status (Tables 6 and 7); therefore, further effort should be made to identify this compound. Some of the other compounds identified here may be useful as indicators of mosquito age and insemination status, which have important demographic and epidemiologic consequences, respectively [58]. For example, because their iCHC quantities did not vary significantly with seasonal treatment or insemination, octacosane and hentriacontane (peaks 6 and 13) appear to be the most reliable biomarkers for age determination*.* This finding may represent a significant contribution, as it has potential to facilitate characterization of the age structure of a mosquito population, and thus the population’s vectorial capacity [59]. Nonacosane and hentriacontane (peaks 9 and 13) have also previously been identified as indicators of age in *An. stephensi*[60]. With respect to insemination status, the peak area of 2,3-dimethylnonadecane (peak 10) did not vary significantly with simulated seasonal treatment or age, making it the most reliable biomarker for distinguishing between mated and virgin populations of nulliparous, non-blood-fed females. An alternative approach for identifying the time of the year when a particular mosquito has eclosed would be to use pentacosane and heptacosane (peaks 3 and 5). However, this would first require determination of the mosquito’s age, since a significant age-by-treatment interaction means that treatment effects are more clearly exhibited in older mosquitoes (Figure 7). The iCHCs measured in this study and provided in Tables 6 and 7 could potentially be used as a key for determining different aspects of *An. gambiae* population structure in the Sahel, provided that these results are consistent in field populations. However it is important to acknowledge that all of our observations concerning CHCs apply only to nulliparous non-blood-fed females and that these patterns could differ for blood-fed, parous females.

## Conclusions

This work shows that *An. gambiae* (M-form) responds to a modest change in photoperiod and possibly relative humidity, with photoperiod inducing morphological changes and possibly shaping initial CHC composition, while the combination of photoperiod and relative humidity affected total CHC amount and possibly its composition later in life. Whereas adult morphological characteristics appear to be fixed and predetermined during the larval and/or pupal stage, CHC amount and composition appear to be dynamic traits that could change during the adult stage. Overall, the morphological and chemical changes observed in this study are consistent with our expectations of these traits constituting adaptations to dry ambient conditions. These changes may or may not reflect aestivation. More functional analyses of the capacity of the mosquitoes reared under dry-season conditions to survive longer and withstand desiccation compared with those reared under wet-season conditions would be needed and have already begun.

This study provides data useful in the assessment of malaria-vector population structures, including putative biomarkers for dry-season-adapted mosquitoes, age, and insemination status*.* Increased understanding of the dry-season survival mechanisms of *An. gambiae* in semi-arid regions could benefit vector control efforts by identifying weak links in the transmission cycle of malaria*.* Specifically, the characteristics distinguishing dry- and wet-season mosquito populations identified in this study may be useful in indicating the time of the year when a particular mosquito has eclosed and relative age of *An. gambiae* populations. Identification of mosquitoes displaying dry-season characteristics during the early wet-season could indicate that they originated early in the dry period and survived the dry season via aestivation. In such a case, efforts should be made to identify these aestivation sites. This, in turn, will enable the application of mosquito control measures in a focused, effective, and environmentally sustainable manner that could, as demonstrated by Adamou *et al.*[6], substantially impact the subsequent mosquito generation population size thereby reducing malaria transmission.

## Abbreviations

GC-MS: Gas chromatography mass spectrometry; iCHC: Standardized individual cuticular hydrocarbons; LP-HRH: Long photoperiod, high relative humidity; SI: Spiracular index; SP-LRH: Short photoperiod, low relative humidity; tCHC: Standardized total cuticular hydrocarbons; t_R_a: Mean n-alkane retention time.

## Competing interests

The authors declare that they have no competing interests.

## Authors’ contributions

KW conducted the experiments, did the analyses, and wrote the majority of the manuscript. TL conceived the basic research idea, contributed to the design of the study, and hosted the experimental stage of the study at the NIH. DH assisted in design, rearing, treatment, and collection of specimens at the NIH. NC supervised the GC analysis at UNCG. BE assisted in conducting the GC analysis at UNCG. GW was the supervisor of KW and contributed to the design of the study, data analyses, and manuscript writing. All authors read and approved the final version of the manuscript.
